# Encapsulation of Beetroot Pomace Extract: RSM Optimization, Storage and Gastrointestinal Stability

**DOI:** 10.3390/molecules21050584

**Published:** 2016-04-30

**Authors:** Vesna Tumbas Šaponjac, Jasna Čanadanović-Brunet, Gordana Ćetković, Mirjana Jakišić, Sonja Djilas, Jelena Vulić, Slađana Stajčić

**Affiliations:** Faculty of Technology, University of Novi Sad, Bulevar cara Lazara 1, Novi Sad 21000, Serbia; jasnab@uns.ac.rs (J.C.-B.); cetkovic@tf.uns.ac.rs (G.C.); jakisicmirjana@gmail.com (M.J.); sdjilas@tf.uns.ac.rs (S.D.); jvulic@uns.ac.rs (J.V.); sladja@uns.ac.rs (S.S.)

**Keywords:** beetroot, polyphenols, betalains, encapsulation, RSM, storage, digestion

## Abstract

One of the great problems in food production are surplus by-products, usually utilized for feeding animals and for preparation of dietary fibre or biofuel. These products represent potential sources of bioactive antioxidants and colour-giving compounds which could be used in the pharmaceutical industry and as food additives. In the present study beetroot pomace extract was encapsulated in soy protein by a freeze drying method. Process parameters (core: wall ratio, extract concentration and mixing time) were optimized using response surface methodology (RSM) in order to obtain the optimum encapsulate (OE) with the highest polyphenol encapsulation efficiency (EE) and radical scavenging activity on DPPH radicals (SA). Using the calculated optimum conditions, the EE (86.14%) and SA (1668.37 μmol Trolox equivalents/100 g) of OE did not differ significantly (*p* < 0.05) from the predicted ones. The contents of total polyphenols (326.51 mg GAE/100 g), flavonoids (10.23 mg RE/100 g), and betalains (60.52 mg betanin/100 g and 61.33 mg vulgaxanthin-I/100 g), individual content of phenolic compounds and betalains by HPLC, and the ability to reduce Fe^3+^ ions, *i.e.*, reducing power (394.95 μmol Trolox equivalents/100 g) of OE were determined as well. During three months of storage at room temperature, polyphenol retention was much higher (76.67%) than for betalain pigments, betacyanins (17.77%) and betaxanthins (17.72%). *In vitro* digestion and release of phenolics from OE showed higher release rate in simulated intestinal fluid than in gastric fluid. These results suggest encapsulation as a contemporary method for valorisation of sensitive bioactive compounds from food industry by-products.

## 1. Introduction

Vegetables and fruits are known to contain components, such as vitamins, essential minerals, antioxidants, and prebiotics (fibres), with several types of health-promoting actions, and most of these have been evaluated in intervention studies [1]. Raising awareness among consumers about functional foods demands investigation of new bioactive compounds that can be used in the production of functional products. Therefore, much attention has been paid to the natural compounds, such as polyphenols, carotenoids, peptides, sterols or polyunsaturated fatty acids (PUFAs), and their associated bioactivities [2,3]. Functional foods include all ranges of food compounds such as vitamins, mineral supplements, herbs, phytochemicals, and probiotics, which are tied up with disease prevention and health promotion [4].

Nowadays, waste management is an environmental problem on a daily basis. Residues from processing of fruits and vegetables are increasingly used as a source of bioactive compounds such as polyphenols, tocopherols, carotenoids and many others. These phytochemicals from waste materials deriving from agro-industrial production may be used as functional food ingredients and as natural antioxidants to replace their synthetic equivalents that have experienced growing rejection [5,6,7,8].

Due to its antioxidant capacity, beetroot is one of the 10 most powerful vegetables [9]. Beetroot contains a significant amount of phenolic acids: ferulic, protocatechuic, vanillic, *p*-coumaric, *p*-hydroxybenzoic and syringic acids [10]. In our previous investigations, we showed that beetroot pomace extract has a high content of bioactive compounds, phenolics and betalains, and possesses important antioxidant and antiproliferative activities [5,9,11,12]. Betalains are water-soluble nitrogenous pigments, which comprise two main groups, red coloured betacyanins and yellow betaxanthins and they have major role like free radical scavengers that prevent active oxygen-induced and free radical-mediated oxidation of biological molecules [13].

Bioactive compounds, such as vitamins, probiotics, minerals, polyphenols, omega-3-fatty acids, and phytosterols are sensitive to oxygen, light, heat, and water [14]. One of the effective methods to preserve the bioactive compounds is microencapsulation [15]. Microencapsulation is a technology for packing solids, liquids or gaseous materials in miniature, sealed capsules to release their contents at controlled rates under specific conditions. Shahidi and Han [16] reported that microencapsulation protects the core from adverse environmental conditions, improve shelf life of a product and promote controlled release.

Soy protein isolate (SPI) made from soybeans is an inexpensive readily available protein that is frequently incorporated into foods and supplements. Soy proteins have functional properties which are suitable for encapsulation, such as solubility, the ability to absorb water and oil, emulsion stabilization, gelation, foaming agents, ability to form the quality film and good organoleptic properties [17]. In comparison with animal proteins, soy protein showed hypocholesterolemic effect, decrease the activity of lipogenic enzymes and reduce body fat [18].

The aim of this study was to optimize the beetroot pomace extract encapsulation by freeze drying using soy protein isolate (SPI) as a carrier. Optimization of encapsulation process variables, *i.e.*, wall to core ratio, extract concentration and mixing time, was carried out taking into account polyphenol encapsulation efficiency and free radical scavenging activity of the encapsulates as responses, using response surface methodology (RSM). The powdered encapsulate obtained under optimum conditions (OE) was characterised for bioactives (polyphenols, flavonoids and betalains) content, free radical scavenging activity, reducing power, storage stability and *in vitro* release of polyphenols during simulated digestion.

## 2. Results and Discussion

### 2.1. Characterization of Beetroot Pomace Extract

For characterizing beetroot pomace extract the chosen representative indices were total phenolics, total flavonoids, total betacyanins, and total betaxanthins content (determined spectrophotometrically), individual phenolics and betalains (HPLC characterization) as well as free radical scavenging activity on DPPH^●^ (SA) and reducing power (RP). The results are presented in Table 1.

From the results presented in Table 1 it is evident that total polyphenols, determined by Folin-Ciocalteu assay, are the dominant bioactive compounds in the beetroot pomace, and that flavonoids represent a small share (5.32%) of the total polyphenols present in the pomace. Also, the betacyanin content was similar to the content of betaxanthins, without significant difference (*p* < 0.05). Kujala *et al.* [10] have reported that the content of polyphenolic compounds in beetroot vary from 4.2 to 15.5 mg of GAE/g of DW, depending on which part of the beet is used for extraction. Vulić *et al.* [5] tested pomace from various varieties of beetroot, ‘Cardeal-F1’, ‘Egyptian’, ‘Bicor’ and ‘Kestrel’. They reported that the contents of phenolics in beetroot pomaces varied from 1.87 to 11.98 mg/g DW. Orhan *et al.* [19] suggest that the genotype, location and techniques of cultivation, as well as differences in the maturity of the plant have an effect on the content of phenolic compounds. Also, external factors such as light, temperature, presence of nutrients in the soil can affect phenylpropanoid metabolism. Caldwell *et al.* [20] have reported that the content of flavonoids depended on UV radiation and the concentration of carbon dioxide. The betacyanin and betaxanthin concentration ratio usually ranges from 1 to 3 and depends mainly on beet varieties, as well as on the respective technology of juice or extract production. The average amount of betalains in beetroot has been estimated as 1000 mg/100 g of total solids, or 120 mg/100 g of fresh weight [21]. Georgiev *et al.* [22] found that betalain contents in the beetroot products were substantial differences in both their betalain concentrations and betacyanin/betaxanthin (BC/BX) ratios. Hairy root extracts yielded 47.11 mg of betalains/g dry extract (16.33 mg betacyanins/g dry extract and 30.78 mg betaxanthins/g dry extract),whereas intact beetroot plant extracts contained 39.76 mg/g dry extract of betalains (20.75 mg betacyanins/g dry extract and 19.01 mg betaxanthins/g dry extract). Vulić *et al.* [5] reported that betalain content in beetroot pomace ranged from 0.75 for cv. ‘Egyptian’ to 3.75 mg/g DW for cv. ‘Bicor’. Eder [23] reported that beetroot pomace extracts had higher betacyanin contents (6.28% in ‘Kestrel’ pomace extract to 18.77% in ‘Bicor’ pomace extract) than current commercially used red beetroot colourants (0.4%–1.0% betacyanins), which makes them a good alternative for use in food production. Compared to the literature data presented here, our results show lower values of total polyphenols and betalains, which is understandable since we tested pomace extract. However, our results are in accordance with the reports of Vulić *et al.* [5], investigating the same material (pomace). Total polyphenols reported in our study are somewhat lower, while betalains are in the same range. Further, the characterization of individual phenolic compounds and betalains was conducted by HPLC analysis. It was revealed that catechin is the dominant phenolic compound and betanin the dominant betalain. HPLC data were in agreement with total contents of polyphenols and betalains determined spectrophotometrically. 

Georgiev *et al.* [22] reported high antioxidant activities of extracts from the beetroot cv. ‘Detroit dark red’. The % inhibition values reported were 90.7% for hairy root cultures and 14.2% for intact plants. Kugler *et al.* [24] reported that beetroot has high antioxidant capacity due to betacyanin betanin in combination with phenolic compounds. Vulić *et al.* [12] used ESR spectroscopy to investigate the free radical scavenging activity of beetroot pomace extract using DPPH^●^, ^●^OH and O_2_^●−^. It was concluded that beetroot pomace extract was more effective at hydroxyl and DPPH radical scavenging than on superoxide anion radicals.

### 2.2. Optimization of Beetroot Pomace Extract Encapsulation

The encapsulation of beetroot pomace extract was optimized using RSM, planning the experiments with Box-Benkhen design (Table 2). Presented variables (X1, X2, and X3) where combined in 15 different encapsulation experiments following the measurements of EE and SA as responses. In our previous study sour cherry pomace extract was encapsulated in whey and soy protein using the same technique, where the final ratio between coating material and extract diluted with water was 167 g/L [18]. The final values of encapsulation parameters, *i.e.*, core:wall ratio and extract dilution, have to consider water absorbing capacity of coating material as well as the contents of bioactive compounds in the sample to be encapsulated in order to obtain reasonably enriched powder. Ramiréz *et al.* [25] have examined the influence of wall–core ratio on encapsulation process using freeze-drying at values 0, 50 and 100. Ezhilarasi *et al.* [26] have encapsulated 66.6 g of concentrated *Garcinia cowa* fruit extract and 46.7 g of water in 20 g of wall material (whey protein isolate, maltodextrin and their mixtures), which makes the final wall–core ratio around 170 g/L, stirring for 30 min before freeze-drying. Similarly, *Averrhoa carambola* pomace extract was encapsulated in maltodextrin with spray- and freeze-drying using 1:10, 1:15 and 1:20 core:coating material ratios, which is similar to our experimental range (1:6.67, 1:10 and 1:20, *i.e.*, 50 g/L, 100 g/L and 150 g/L) [27]. The actual values were chosen from the preliminary studies, based on literature survey [18,25,26,27,28,29] and, and the corresponding coded values of three independent variables are given in Table 2. The results of encapsulation parameters (core–wall ratio, extract dilution and mixing time) influence on beetroot pomace extract encapsulation efficiency (EE) and encapsulate radical scavenging activity (SA) are given in Table 2.

**Figure 1:**
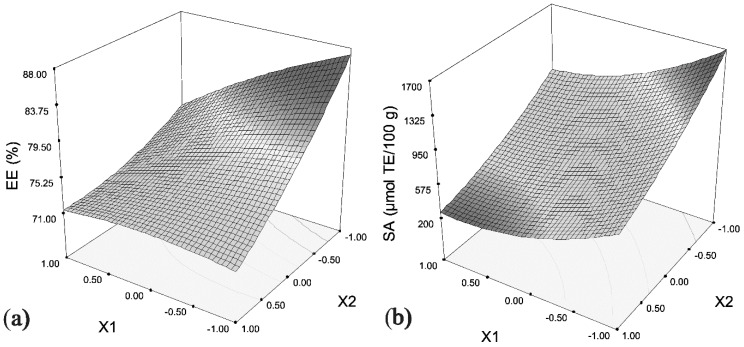
The influence of encapsulation parameters on (**a**) EE of beetroot pomace polyphenols and (**b**) SA of beetroot pomace extract encapsulates.

From Table 2 it can be seen that experimental values of EE ranged from 60.26% (experiment 8), up to 85.03% (experiment 9). The experimental values for SA were in the range from166.96 μmol TE/100 g for the experiment 12, to 1619.22 μmol TE/100 g for the experiment 1. Moreover, the experimental values for SA were highly correlated (r = 0.94) to total polyphenol contents inside the core of encapsulate (CPC values, data not shown). Microencapsulation of polyphenols depends greatly on the carrier and technique employed. It has been reported that encapsulation efficiency of polyphenols from pomegranate juice and extract in soy protein and maltodextrin (PJ-SPI, PJ-MD, PE-SPI and PE-MD) using spray-drying were in the range of 36.6%–62.8%, 51.4%–82.8%, 52%–81.5% and 52.9%–82.8%, respectively [30]. Microencapsulation of cactus pear juice polyphenols in soy protein isolate, maltodextrin and inulin and their mixtures by spray-drying resulted in efficiency ranging from 72.8% to 86.5% [31]. Figure 1 shows the influence of independent variables (X1, X2, X3) on EE and SA of encapsulates obtained in experiments 1–15.

Based on the EE and SA values obtained in optimization experiments (Table 2), in order to find the optimal conditions for encapsulation of beetroot pomace extract in soy protein, single and multi-response optimization was carried out and results are reported in Table 3.

As a first criterion for single response optimization maximum of the response surface that shows the influence of tested parameters on response EE (Figure 1a) was set. It was found that a low core–wall ratio (50.50 g/L), low extract dilution (0.22) and medium mixing time (14.1 min) is needed to ensure maximum encapsulation efficiency (86.16%). Single response optimization was performed to optimize the response SA as well (Figure 1b). According to this optimization, the optimal sample, *i.e.*, the encapsulate with maximum SA (1676.17 µmol/100 g) can be obtained by using relatively low of core–wall ratio (51 g/L), almost no extract dilution (0.04) and medium mixing time (16 min). For selecting optimal sample (OE) which will be tested further a multi response optimization was employed, where both responses, *i.e.*, EE and SA are considered at the same time and the obtained result represents the optimal conditions that provide maximum values of both responses. The optimal wall–core ratio and extract dilution for obtaining enriched soy proteins with the highest EE and the highest SA on DPPH^●^ are the same as for single optimization of SA, while mixing time should be slightly longer (18.60 min).

Saikia *et al.* [27] examined the encapsulation efficiency of the pomace of *Averrhoa carambola* and found that the encapsulation efficiency was much higher in freeze-dried samples (78%–97%) than in samples obtained by spray-drying methods (63%–79%). The variation in efficiency could be due to susceptibility of some phenolic acids to destruction during the application of heat in the encapsulation process by spray-drying [32].

### 2.3. Characterization of the OE

Using the parameters obtained in multi response optimization (Table 3) and under the same freeze-drying conditions used in experiments 1–15, the OE was obtained. The OE was characterized in terms of the content of bioactive compounds and biological activity, in order to confirm the optimization model. Also, the storage stability and release of the encapsulated polyphenol content during *in vitro* digestion of optimal sample was tested.

#### 2.3.1. Bioactive Compounds and Biological Activity

The content of total polyphenolic compounds, flavonoids and betalains, (spectrophotometrically), individual phenolics and betalains (HPLC characterization) in OE, as well as its radical scavenging activity (SA) and reducing power (RP) are reported in Table 4.

Similarly to the beetroot pomace extract, flavonoids have a small share (3.13%) in total polyphenol content of encapsulate determined by Folin-Ciocalteu assay, and the content of betaxanthins (61.33 mg VE/100 g encapsulate) is similar to the content of betacyanins (60.52 mg BE/100 g encapsulate), with no significant difference (*p* < 0.05). On the other hand, it is evident that the content of polyphenols is much higher than betalains, compared to their ratio in beetroot pomace extract (Table 1), pointing to higher bonding affinity of soy proteins towards these compounds. HPLC analysis of OE encapsulate showed that phenolic contents are in accordance with spectrophotometrical data on total polyphenol contents and betalains in OE. Likewise in the beetroot pomace extract, catechin was the most abundant phenolic compound and betanin was the dominant betalain in OE. Encapsulation efficiency of the optimal sample was 86.28%, which is in accordance with the result obtained in a multi response optimization (Table 3), with no statistically significant difference (*p* < 0.05). Predicted SA values obtained in a multi response optimization (Table 3) are also in accordance with the values obtained for OE, without significant difference (*p* < 0.05).

Janiszewska [33] encapsulated beetroot juice using Arabic gum and maltodextrin as a carrier. The highest content of yellow pigments was found for maltodextrin (57–61 mg/100 g) while the content of betanin was higher in Arabic gum encapsulates (123–129 mg/100 g). Robert *et al.* [30] encapsulated pomegranate juice in maltodextrin and soy protein isolate by spray-drying technique. The polyphenols encapsulating efficiency was significantly better when soy protein was used. Experimental data showed that encapsulation in maltodextrin greatly improves the stability of betalains to high degree and maintenance of the antiradical properties [34]. The antioxidant ability of certain compounds is associated with their reducing power; thus the reducing power may serve as a significant indicator of potential antioxidant activity [35]. The presence of reducers (*i.e.*, antioxidants) causes the reduction of the Fe^3+^/ferricyanide complex to the ferrous form. Therefore, spectrophotometrical measurement of the formation of Perl’s Prussian blue at 700 nm can monitor the Fe^2+^ concentration. The results on reducing power demonstrate the electron donor properties of beetroot pomace extracts, thereby neutralizing free radicals by forming stable products [5].

#### 2.3.2. Storage Stability Studies

**Figure 2:**
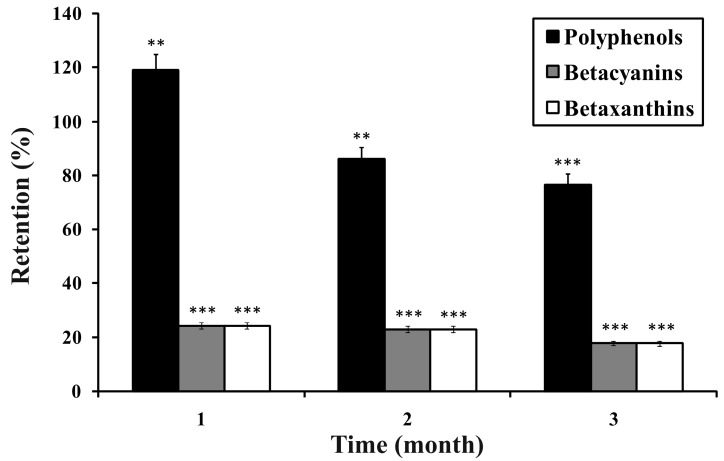
Polyphenol and betalain stability in OE during storage. Significant increase/decrease of retention at the level ** *p* < 0.01 or *** *p* < 0.001. Results are expressed as mean ± SD of three independent experiments.

The results of testing the OE powders’ polyphenol and betalain shelf life are presented in Figure 2. During the first month the polyphenol content in OE has slightly increased (for 19.08%), while after three months the content of polyphenols in encapsulate has decreased by 23.33% of the initial content.

However, the content of betalains has decreased to much higher extent. The retention of both pigments, *i.e.*, violet betacyanins and yellow betaxanthins, decreased more rapidly during the first month (to 24.25% and 24.17%, respectively). During the second and third month the retention of betacyanins and betaxanthins did not change significantly (*p* < 0.05). Both pigments have shown the same trend of degradation during storage period, with no significant difference in their retention (*p* < 0.05). These results confirm that soy proteins show higher affinity towards polyphenols than for betalains. Also, betalains are more sensitive and prone to degradation during storage, which includes the influence of exogenous factors like light, oxygen *etc.* These results are in accordance with the findings of other authors. Saénz *et al.* [36] have followed the content of polyphenols and betalains in pulp and ethanolic extracts of the cactus pear (*Opuntia ficus-indica*) encapsulated in maltodextrin or inulin during 44 days of accelerated storage. It was determined that the retention of polyphenols increased during the first 20 or 44 days of storage (up to 158%), depending on the encapsulate sample. In some samples a small decrease of polyphenol retention was found after 44 days (up to 77%). On the other hand, after 44 days the retention of betalains, betacyanin and indicaxanthin decreased up to 59% and 50%, respectively. It has been reported that at elevated temperatures isomerisation and decarboxylation of betacyanins occur [37]. Betacyanins from *Amaranthus* were used to colour jelly, ice cream and beverage with good colour stability at low temperatures after 12 or 18 weeks, but with inferior stability at room temperature [38]. Vergara *et al.* [39] reported complete disappearance of betanin from cactus pear extract encapsulated in Capsul^®^ (modified starch-based encapsulating agent) after 160 days of storage at 60 °C.

#### 2.3.3. *In Vitro* Digestion of OE in the Intestinal Fluids

**Figure 3:**
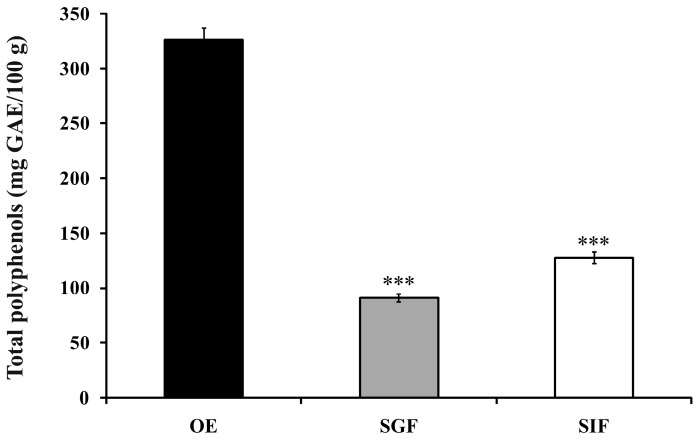
*In vitro* gastrointestinal release of polyphenols in simulated gastric fluid (SGF) and simulated intestinal fluid (SIF) after *in vitro* digestion of optimal encapsulate (OE) (*** *p* < 0.001; One way analysis of variance, compared to the control, OE). Results are expressed as mean ± SD of three independent experiments.

*In vitro* digestion and release of the encapsulated polyphenol content from OE was determined by simulating the digestion conditions in gastric and intestinal fluids. Content of the total polyphenols was determined by method of Folin-Ciocalteu and the results are shown in Figure 3.

Test results showed that the total polyphenol content after simulation of encapsulate gastric digestion was 90.85 mg GAE/100 g, while after its intestinal digestion the content of total polyphenols was 127.45 mg GAE/100 g. From these results it can be concluded that the release of polyphenolic compounds from encapsulate was more intense in intestinal fluid than in gastric fluid (39.03% and 27.82% of the initial total polyphenol value, respectively) during the *in vitro* digestion.

Flores *et al.* [40] followed the *in vitro* release of polyphenolic compounds in blueberry extracts encapsulated in gum Arabic and whey proteins, obtained by spray-drying. It was observed that these two matrixes showed different behaviour. Gum arabic as a matrix releases polyphenols more easily in gastric fluid than in intestinal fluid. Soy protein was slowly releasing polyphenols in gastric acid, while a high level of polyphenols and antioxidant activity was found in the intestinal fluid. On the other hand, Saikia *et al.* [27] have shown that the release of polyphenols from encapsulated *Averrhoa carambola* pomace extract on maltodextrin in gastric fluid at pH 1.2 was more significant than in the intestinal fluid at pH 6.8. Similar results have been obtained in the study of Işık *et al.* [41] following *in vitro* digestion of onion and apple pomace extract encapsulated in modified starch and pea proteins. It can be summarized that the behaviour of encapsulates during *in vitro* digestion depends on the composition of matrix in which the encapsulation was carried out, and their resistance and susceptibility to digestive enzymes, as well as the conditions in the gastrointestinal tract such as pH [42,43].

## 3. Material and Methods

### 3.1. Chemicals and Instruments

Folin-Ciocalteau reagent, 2,2-diphenyl-1-picrylhydrazyl radical (DPPH^●^), Trolox, trichloroacetic acid, pancreatin, pepsin and all standards for HPLC analysis (phenolics and betalains) were purchased from Sigma Chemical Co. (St Louis, MO, USA), ferric chloride was obtained from J.T. Baker (Deventer, Holland), and sodium nitrite from LACH-NER (Brno, Czech Republic). Other chemicals and solvents used were of the highest analytical grade. Distilled water was produced using water purification system DESA 0081 Water Still destilator (POBEL, Madrid, Spain). Soy protein isolate was purchased from “Macrobiotic Prom” company (Belgrade, Serbia). Absorbances in spectrophotometrical assays were measured on a Multiskan GO microplate reader (Thermo Fisher Scientific Inc., Waltham, MA, USA) and UV-1800 spectrophotometer (Shimadzu, Kyoto, Japan). For HPLC analysis a Shimadzu Prominence chromatographic system was used, which consisted of LC-20AT binary pump, CTO-20A thermostat and SIL-20A autosampler connected to the SPD-20AV UV/Vis detector (Shimadzu, Kyoto, Japan).

### 3.2. Plant Material

Beetroot (*Beta vulgaris* L., cv. ‘Bicor’) was obtained at a local supermarket. Roots of the beet were washed, cut into pieces and blended in a laboratory blender (model Neo SK-400, TCL King Electrical Appliances Co., Ltd., Huizhou, China). Beetroot pomace was separated from juice by vacuum filtration. Pomace was freeze-dried and stored at −20 °C until analysis. 

### 3.3. Pomace Extraction Procedure

Sample of freeze dried beetroot pomace (25 g) was extracted using 50% aqueous ethanol (250 mL) with 0.5% acetic acid on ultrasonic bath at room temperature for 30 min. Macerate was filtered by vacuum filtration using Whatman filter paper Ø47 mm, concentrated under reduced pressure by rotary evaporation set at 40 °C until removal of ethanol and immediately mixed with soy protein isolate as a carrier and encapsulated. 

### 3.4. Encapsulation Optimization

In order to encapsulate beetroot pomace extract experimental design RSM was used to optimize encapsulation process parameters. The experimental design adopted was a Box-Behnken design for three variables at three levels. The three independent variables (encapsulation process conditions) were: core:wall ratio (X1), extract dilution (X2) and mixing time (X3). The coded values of the independent variables were –1, 0 and 1. The actual values were chosen from the preliminary studies, based on literature survey [18,25,26,27,28,29] and taking into account water absorbing properties of soy protein isolate and total polyphenol content in beetroot pomace extract in order to obtain sufficient levels of bioactive compounds in the final product (encapsulate), and the corresponding coded values of three independent variables are given in Table 2. The complete design consisted of 15 experimental points, which included three replicates of central point. The homogenized mixtures were frozen at −18 °C for 3 h and then freeze dried at −40 °C for 48 h to ensure complete drying [44], yielding free-flowing powders, coloured in different shades of purple. The encapsulate powders were packed in airtight containers and stored at 4 °C until further use.

### 3.5. Total Phenolic Content

The total polyphenol contents in beetroot extract, encapsulates as well as in simulated digestion fluids were determined spectrophotometrically by Folin-Ciocalteau method adapted to microscale [45]. Briefly, the reaction mixture was prepared in 96 well microplate by mixing 15 µL of extract, 170 µL of distilled water, 12 µL of the Folin-Ciocalteu’s reagent (2 M) and 30 µL of 20% sodium carbonate. After 1 h, the absorbance at 750 nm was obtained using distilled water as blank. Results were expressed as gallic acid equivalents (GAE) per 100 g sample.

#### Phenolic Content in the Core (CPC) and Surface (SPC) of Encapsulate and Encapsulating Efficiency (EE)

For CPC and SPC the sample extraction procedure by Sáenz *et al.* [30] was used. For the core phenolic content, 100 mg of sample was dispersed in 1 mL ethanol, acetic acid and water (50:8:42). The mixture was then vortexed for 1 min, centrifuged for 2 min, and the supernatant was separated. Similarly, in case of SPC, 100 mg of sample was dispersed in 1 mL of ethanol and methanol (1:1) mixture. The mixture was vortexed for 1 min, centrifuged for 2 min, and the supernatant was separated. The CPC and SPC were determined by Folin–Ciocalteu method described above and results were expressed as mg of gallic acid equivalent (mg GAE/100 g encapsulate). The encapsulating efficiency was determined by using the given equation:

EE (%) = ((CPC − SPC)/CPC) × 100
(1)

where CPC is the phenolic content inside the core of the encapsulate; SPC is the surface phenolic content. The corrections for interfering substances originating from soy protein has been made by simultaneously preparing control samples replacing encapsulate extract with matching concentration of soy protein extract prepared in the same way.

### 3.6. Total Flavonoids Content

Total flavonoid contents in beetroot extract and OE were determined by means of the aluminium chloride colourimetric assay [46] adapted for 96 well microplate. Flavonoids from beetroot pomace or optimal encapsulate (0.2 g) were extracted in 2 mL of extraction medium (70% (*v*/*v*) methanol, 5% [v/v] acetic acid and 25% (*v*/*v*) distilled water) at room temperature for 60 min. The resulting solution was filtered through Whatman paper No. 4 and filtrate volume adjusted to 10 mL. The probes were prepared in microplate wells by mixing: 5 mL of extract, 1 mL of distilled water and 2.5 mL of AlCl_3_ solution (26.6 mg AlCl_3_ × 6H_2_O and 80 mg CH_3_COONa dissolved in 20 mL distilled water). A blank probe was prepared by replacing AlCl_3_ solution with distilled water. The absorbance of probes and blank probe were measured immediately at 430 nm. Measurements included corrections for interfering substances originating from soy protein in OE by preparing control samples replacing encapsulate extract with matching concentration of soy protein extract prepared in the same way. Results were expressed as rutin equivalents (RE) per 100 g sample.

### 3.7. Determination of Betalain Content

The betalains (betacyanins and betaxanthins) pigment contents in beetroot pomace extract and OE were measured according to von Elbe [47] adapted for 96 well microplate. OE sample was prepared in the same way as for determination of CPC. In microplate well 10 or 20 µL of beetroot pomace extract or encapsulate extract was mixed with 240 µL or 230 µL of phosphate buffer (0.05 M, pH 6.5). Phosphate buffer was used as blank. The wavelengths of 535 and 476 nm were used for betacyanin and betaxanthin analysis, respectively, and 600 nm for correction. Absorbances of betanin and vulgaxanthin-I were calculated using the following equations:

x = 1.095 × (a − c)
(2)

y = b − z − x/3.1
(3)

z = a − x
(4)

where a is absorbance at 538 nm, b is absorbance at 476 nm, c is absorbance at 600 nm, x is absorbance of betanin corrected for coloured impurities, y is absorbance of vulgaxanthin-I corrected for coloured impurities and z is absorbance of impurities. Betanin and vulgaxanthin-I concentrations in beetroot pomace extract and optimal encapsulate was calculated using the equation:

C (mg/100 mL) = x(y) × F × 1000/A^1%^ (5)

where F is dilution factor (25 or 12,5) and A^1%^ is absorbance coefficient (1120 for betanin, 750 for vulgaxanthin). The betacyanins content was expressed as mg betanin equivalents per 100 g of sample (mg BE/100 g), and the betaxanthins content was expressed as mg vulgaxanthin-I equivalents per 100 g of sample (mg VE/100 g).

### 3.8. HPLC Analysis

All analyte solutions and solvents were filtered prior to analysis through 0.45 µm (pore size) membrane filters (Millipore, Bedford, MA). OE sample was prepared in the same way as for determination of CPC. For analysis of phenolic compounds chromatograms were recorded using different wavelengths for individual phenolic compounds: 280 nm for hydroxybenzoic acids (gallic, protocatechuic, vanillic and syringic acid), catechins (catechin, epicatechin and epicatechin gallate), and ellagic acid, 320 nm for hydroxycinnamic acids (caffeic, chlorogenic, coumaric, ferulic, isoferulic, synapic and rosmarinic acid), and 360 nm for flavonoids (quercetin, rutin, luteolin, myricetin and kaempferol). Separation was performed on a Luna C-18 RP column, 5 µm, 250 mm × 4.6 mm (Phenomenex, Torrance, CA, USA) with a C18 guard column, 4 mm × 30 mm (Phenomenex, Torrance, CA, USA). Two mobile phases, A (acetonitrile) and B (1% formic acid) were used at flow rates of 1 mL/min with the following gradient profile: 0–10 min from 10% to 25 % A; 10–20 min linear rise up to 60% A, and from 20 min to 30 min linear rise up to 70% A, followed by 10 minutes reverse to initial 10% A with additional 5 min of equilibration time. Reference substances were dissolved in 50% methanol.

For analysis of betalains solvent gradient was performed by varying the proportion of solvent A (acetonitrile) to solvent B (0.5% HCOOH in water) at flow rates 0.5 mL/min as follows: initial 100% B; linear gradient to 100% B in 3 min; linear gradient to 25% B in 25 min; linear gradient to 25% B in 30 min; linear gradient to 100% B in 35 min. The set time of recording chromatograms and spectra was 35 min, while post-running time was 5 min. The column temperature was 30 °C. The chromatograms were plotted at 538 and 477 nm. Betalain compounds in samples were identified by matching the retention time and their spectral characteristics against those of prepared standards. External standards (isolated from the extract) were used for quantification. External standards were isolated using paper chromatography using isopropanol-ethanol-water-acetic acid (6:7:6:1) as mobile phase, according to Sherma and Fried [48].

### 3.9. Radical Scavenging Activity (SA) by DPPH^●^ Assay

Samples were prepared in the same way as for determination of CPC. The assay was performed spectrophotometrically in a 96-well micro-plate reader, according to Girones-Vilaplana *et al.* [49]. 250 µL of DPPH*^•^* solution in methanol (0.89 mM) was mixed with 10 µL of sample in microplate well and left in dark at room temperature. Absorbances were read at 515 nm after 50 min. Methanol was used as blank. The measurements included corrections for interfering substances originating from soy protein by preparing control samples replacing encapsulate extract with matching concentration of soy protein extract prepared in the same way. SA values were calculated using the following equation:

SA (%) = (A_Control_ − A_0_)/A_Control_ × 100 (6)

where A_Control_ is the absorbance of the control reaction, A_0_ initial absorbance of DPPH^•^ solution and A_Sample_ is the absorbance in the presence of extracts. The calibration curve was made with Trolox and results were expressed as μmol of Trolox equivalents per 100 g of sample (μmol TE/100 g).

### 3.10. Reducing Power (RP)

Reducing power was determined by the method of Oyaizu [50] adapted for 96 well microplate. Samples were prepared in the same way as for determination of CPC. In brief, 25 µL of sample, adequately diluted (final absorbance according to the method should be in the range of from 0.2 to 0.8) or 25 µL water (blank test), 25 µL of sodium phosphate buffer pH 6.6 and 25 µL of 1% potassium ferricyanide were mixed, incubated in a water bath for 20 min at 50 °C. After cooling, 25 µL of 10% trichloroacetic acid was added and solutions were centrifuged at 3000 rpm for 10 min. After centrifugation, 50 µL of supernatant was mixed with 50 µL of distilled water and 10 µL of 0.1% ferric chloride in a well of a microplate. Absorbances were measured immediately at 700 nm. The measurements included corrections for interfering substances originating from soy protein by preparing control samples replacing encapsulate extract with matching concentration of soy protein extract prepared in the same way. The calibration curve was made with Trolox and results were expressed as μmol of Trolox equivalents per 100 g of sample (μmol TE/100 g).

### 3.11. Storage Stability Test

For testing the shelf life of encapsulated bioactive compounds room storage conditions were employed [25,51,52,53]. Optimum encapsulate sample (OE) was stored at room temperature (25 °C) in high-density polyethylene bags for three months to determine the effect of time on the stability of total polyphenols and betalain pigments. For that purpose, three portions of 100 mg of OE powder were removed every month, in triplicate. Total polyphenols and betalain pigments were determined by the corresponding methods described above.

### 3.12. In Vitro Simulated Gastric and Intestinal Digestion Release Study

*In vitro* digestion and release of apparent phenolic content by OE was determined by simulation of digestion in gastric and intestinal fluid, according to the method given by Saikia *et al.* [27]. The simulated gastric fluid (SGF) and simulated intestinal fluid (SIF) were prepared as given in U.S. Pharmacopeia [54]. After the treatment with digestive enzymes, both samples of SGF and SIF were analysed for total phenolic content by Folin-Ciocalteu method described in the Section 3.5.

### 3.13. Statistical Analysis

All experiments were run in triplicate. The results represented are means ± standard deviation (± SD, *n* = 3). Statistical analyses were done by using Origin 7.0 SRO software package (OriginLab Corporation, Northampton, MA, USA, 1991–2002) and Microsoft Office Excel 2010 software. Significant differences were calculated by ANOVA (*p* < 0.05). For optimization experiments, the models quality was determined by analysis of variance (ANOVA) of regression. Calculations and graphs, single and multi-response optimization was conducted using Design-Expert^®^ Version 7.0.0, Stat-Ease, Inc., Minneapolis, MN, USA (2005).

## 4. Conclusion

Beetroot pomace, a waste in the production of beetroot juice, is potentially a powerful source of bioactive compounds and therefore could be utilized further in the development of functional food products. Isolated bioactive compounds can be used in encapsulated form, enabling their easier handling, higher stability and controlled release. For encapsulation of beetroot pomace extract soy protein isolate was chosen as cheap and readily available source of proteins. Optimization of the encapsulation process parameters is a very important step to conduct the efficient entrapment of valuable bioactive compounds. RSM method presented in this paper showed good correlation between predicted and actual characteristics of the optimal encapsulate obtained under the optimal conditions obtained by RSM model. The release of polyphenolic compounds from beetroot pomace extract encapsulate was more intense in simulated intestinal fluid than in gastric fluid. Future research on encapsulation of beetroot pomace extract will be based on its incorporation in model and real food systems as functional food development strategy. Likewise, human studies targeting the bioaccessibility and bioavailability of encapsulated beetroot phytochemicals are required to further explore their biological activities.

## Figures and Tables

**Table 1:** The content of bioactive compounds and antioxidant activity of beetroot pomace.

Bioactive Compounds/Bioactivity	Content (per 100 g Pomace Dry Weight)
Polyphenols ^a^	89.06 ± 4.62
Flavonoids ^b^	4.74 ± 0.16
Betacyanins ^c^	79.22 ± 2.04
Betaxanthins ^d^	80.06 ± 2.06
Gallic acid ^e^	7.45 ± 0.24
Protocatechuic acid ^e^	11.53 ± 0.40
*p-*Hydroxybenzoic acid ^e^	0.78 ± 0.02
Catechin ^e^	53.42 ± 1.53
Epicatechin ^e^	2.09 ± 0.08
Chlorogenic acid ^e^	0.30 ± 0.01
Caffeic acid ^e^	0.18 ± 0.00
Ferulic acid ^e^	0.48 ± 0.02
Sinapic acid ^e^	0.52 ± 0.03
Coumaric acid ^e^	0.34 ± 0.01
Myricetin ^e^	3.52 ± 0.14
Luteolin ^e^	0.03 ± 0.00
Quercetin ^e^	0.22 ± 0.10
Apigenin ^e^	0.10 ± 0.00
Isorhamnetin ^e^	0.35 ± 0.01
Betanin ^e^	88.94 ± 3.57
Vulgaxanthin-I ^e^	71.08 ± 1.33
SA ^f^	59.75 ± 2.09
RP ^f^	206.84 ± 9.81

Data present mean value of three replicates ± SD; ^a^ Expressed as mg gallic acid equivalents (GAE)/100 g; ^b^ Expressed as mg rutin equivalents (RE)/100 g; ^c^ Expressed as mg betanin equivalents (BE)/100 g; ^d^ Expressed as mg vulgaxanthin-I equivalents (VE)/100 g; ^e^ Expressed as mg/100 g; ^f^ Expressed as µmol Trolox equivalents (TE)/100 g.

**Table 2:** Experimental design, encapsulation efficiency and scavenging activity of encapsulates.

Experiment	Wall–Core Ratio (X1, g/L)	Extract Dilution (X2)	Mixing Time (X3, min)	EE (%) ^a^	SA (µmol TE/100 g) ^a^
1	50 (−1)	0 (−1)	15 (0)	84.41 ± 1.56	1619.22 ± 58.64
2	150 (+1)	0 (−1)	15 (0)	77.54 ± 1.49	947.13 ± 29.74
3	50 (−1)	4 (+1)	15 (0)	69.69 ± 4.64	772.19 ± 11.40
4	150 (+1)	4 (+1)	15 (0)	74.18 ± 3.09	339.76 ± 3.96
5	50 (−1)	2 (0)	5 (−1)	82.72 ± 5.50	959.41 ± 18.98
6	150 (+1)	2 (0)	5 (−1)	77.69 ± 5.00	316.30 ± 10.47
7	50 (−1)	2 (0)	25 (+1)	75.40 ± 4.18	998.77 ± 47.09
8	150 (+1)	2 (0)	25 (+1)	60.26 ± 0.32	254.67 ± 24.62
9	100 (0)	0 (−1)	5 (−1)	85.03 ± 2.00	921.69 ± 14.62
10	100 (0)	4 (+1)	5 (−1)	76.36 ± 3.95	223.61 ± 14.17
11	100 (0)	0 (−1)	25 (+1)	78.16 ± 4.17	1077.27 ± 29.22
12	100 (0)	4 (+1)	25 (+1)	64.23 ± 0.15	166.96 ± 19.77
13	100 (0)	2 (0)	15 (0)	75.87 ± 4.75	630.29 ± 24.48
14	100 (0)	2 (0)	15 (0)	75.69 ± 3.46	604.68 ± 32.94
15	100 (0)	2 (0)	15 (0)	76.48 ± 0.90	776.99 ± 0.28

^a^ Results are presented as mean values of three replicates ± SD.

**Table 3:** Single and multi-response optimization of encapsulation parameters.

Optimization	Variable Codes			Variable Values			Optimal Responses	
	X1	X2	X3	X1	X2	X3	EE (%)	SA (µmol/100 g)
Single response (EE)	−0.99	−0.89	−0.09	50.50	0.22	14.1	86.16	-
Single response (SA)	−0.98	−0.98	0.10	51.00	0.04	16.00	-	1676.17
Multi response (EE + SA)	−0.98	−0.98	0.36	51.00	0.04	18.60	86.14	1668.37

**Table 4:** The content of bioactive compounds and bioactivity of OE.

Bioactive Compounds/Bioactivity	Content (per 100 g OE)
Polyphenols ^a^	326.51 ± 3.00
Flavonoids ^b^	10.23 ± 1.22
Betacyanins ^c^	60.52 ± 0.92
Betaxanthins ^d^	61.33 ± 0.92
Gallic acid ^e^	26.54 ± 0.89
Protocatechuic acid ^e^	65.58 ± 2.65
*p-*Hydroxybenzoic acid^e^	30.18 ± 0.02
Catechin ^e^	156.04 ± 1.03
Epicatechin ^e^	3.88 ± 0.05
Chlorogenic acid ^e^	0.55 ± 0.01
Caffeic acid ^e^	0.34 ± 0.00
Ferulic acid ^e^	0.89 ± 0.02
Sinapic acid ^e^	0.97 ± 0.01
Coumaric acid ^e^	0.64 ± 0.01
Myricetin ^e^	6.55 ± 0.09
Luteolin ^e^	0.06 ± 0.00
Quercetin ^e^	0.41 ± 0.08
Apigenin ^e^	0.18 ± 0.00
Isorhamnetin ^e^	0.65 ± 0.01
Betanin ^e^	65.36 ± 2.18
Vulgaxanthin-I ^e^	59.29 ± 2.05
SA ^f^	1655.36 ± 80.98
RP ^f^	394.95 ± 13.05

Data present mean value of three replicates ± SD. ^a^ Expressed as mg gallic acid equivalents (GAE)/100 g; ^b^ Expressed as mg rutin equivalents (RE)/100 g; ^c^ Expressed as mg betanin equivalents (BE)/100 g; ^d^ Expressed as mg vulgaxanthin-I equivalents (VE)/100 g; ^e^ Expressed as mg/100 g; ^f^ Expressed as µmol Trolox equivalents (TE)/100 g.
